# The genome sequence of a soliderfly, *Stratiomys singularior *(Harris, 1776)

**DOI:** 10.12688/wellcomeopenres.23294.1

**Published:** 2024-11-05

**Authors:** Olga Sivell, Duncan Sivell, Ryan Mitchell, Natural History Museum Genome Acquisition Lab, Darwin Tree of Life Barcoding collective, Wellcome Sanger Institute Tree of Life Management, Samples and Laboratory team, Wellcome Sanger Institute Scientific Operations: Sequencing Operations, Wellcome Sanger Institute Tree of Life Core Informatics team, Tree of Life Core Informatics collective, Darwin Tree of Life Consortium

**Affiliations:** 1 Natural History Museum, London, England, UK; 2 Independent researcher, Sligo, County Sligo, Ireland

**Keywords:** Stratiomys singularior, soldierfly, genome sequence, chromosomal, Diptera

## Abstract

We present a genome assembly from an individual female solidierfly, *Stratiomys singularior* (Arthropoda; Insecta; Diptera; Stratiomyidae). The genome sequence has a total length of 715.20 megabases. Most of the assembly is scaffolded into 6 chromosomal pseudomolecules, including the X sex chromosome. The mitochondrial genome has also been assembled and is 15.67 kilobases in length. Gene annotation of this assembly on Ensembl identified 11,614 protein-coding genes.

## Species taxonomy

Eukaryota; Opisthokonta; Metazoa; Eumetazoa; Bilateria; Protostomia; Ecdysozoa; Panarthropoda; Arthropoda; Mandibulata; Pancrustacea; Hexapoda; Insecta; Dicondylia; Pterygota; Neoptera; Endopterygota; Diptera; Brachycera; Stratiomyomorpha; Stratiomyidae; Stratiomyinae; Stratiomyini; *Stratiomys*; *Stratiomys singularior* (Harris, 1776) (NCBI:txid511597).

## Background

*Stratiomys singularior* is a large and distinct species from the family Stratiomyidae, commonly referred to as soldierflies. It has a broad and flattened abdomen which is characteristic for this family. With a body length of 12–15 mm and wing length of 9–12 mm, it is a fairly large species ( Stubbs & Drake, 2014). Black and yellow, it is similar in appearance to other British *Stratiomys* species, however, it can be identified using number of characters. The pale markings on the abdomen of *S. singularior* are relatively small, yellowish or whitish in colour and never meet in the middle of the tergites. Markings in *S. potamida* and *S. chameleon* are more pronounced and brilliant yellow, some of them may cross the width of the abdomen. *Stratiomys longicornis* has indistinct markings on the abdomen but has two dark rings on the hind tibia, compared to one dark ring in *S. singularior* ( Stubbs & Drake, 2014). The thorax of *S. longicornis* has a dense covering of reddish-grey hairs which also sets it apart from the other British *Stratiomys* ( Zeegers & Schulten, 2022). The thorax of *S. singularior* is covered with pale yellow or greyish hairs, the antenna with the first segment about four times as long as the second. The males have hairy eyes, the female eyes are bare ( Stubbs & Drake, 2014).

*Stratiomys singularior* is a widely distributed Palaearctic species, recorded from Armenia, Austria, Belgium, Bulgaria, China, Czech Republic, Denmark, England, Estonia, Finland, France, Germany, Hungary, Iran, Ireland, Italy, Kazakhstan, Lithuania, Mongolia, Netherlands, Norway, Poland, Romania, Russia, Slovakia, Spain, Sweden, Switzerland, Ukraine and Yugoslavia ( Woodley, 2001). In Britain and Ireland, this species is widely distributed across England, southern Wales, and scattered across Ireland ( NBN Trust, 2024). *S. singularior* is on the wing from late May to early September, peaking in July ( Stubbs & Drake, 2014).

The larva has been described by Rozkošný (1982). It is yellowish brown with longitudinal stripes, similar in appearance to larva of *S. longicornis*. It can be separated from other common species by characters given by Stubbs and Drake (2014). The larva is aquatic and can be found in grazing marshes and ditches (also shallow and dominated by algae), muddy washlands and brackish marshes. The larvae of widely varying sizes can be encountered at all seasons, and it has been suggested by Drake (2005) that the species life cycle is likely biannual.

**Figure 1.**
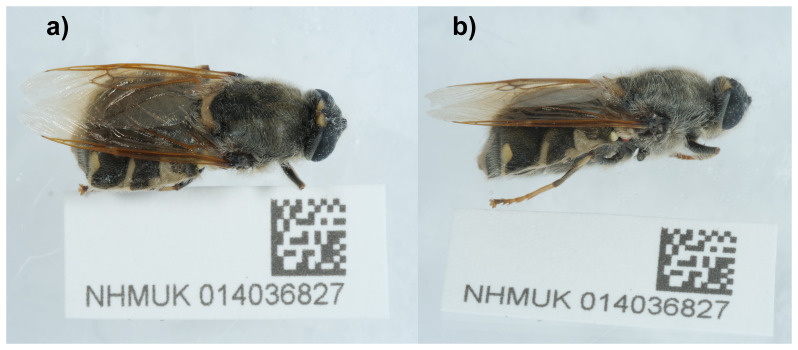
Photographs of the *Stratiomys singularior* (idStrSing1) specimen used for genome sequencing.

The high-quality genome of *Stratiomys singularior* was sequenced from a female specimen (NHMUK014036827; SAMEA11025046) from Cothill Fen National Nature Reserve, England ( Figure 1). It will aid research on taxonomy and biology of the species and the phylogeny of the family Stratiomyidae. The genome was sequenced as part of the Darwin Tree of Life Project, a collaborative effort to sequence all named eukaryotic species in the Atlantic Archipelago of Britain and Ireland.

## Genome sequence report

The genome of an adult female *Stratiomys singularior* was sequenced using Pacific Biosciences single-molecule HiFi long reads, generating a total of 21.27 Gb (gigabases) from 1.83 million reads, providing an estimated 29-fold coverage. Primary assembly contigs were scaffolded with chromosome conformation Hi-C data, which produced 139.44 Gb from 923.43 million reads, yielding an approximate coverage of 195-fold. Specimen and sequencing details are summarised in Table 1.

**Table 1.** Specimen and sequencing data for *Stratiomys singularior*.

Project information			
**Study title**	*Stratiomys singularior*		
**Umbrella BioProject**	PRJEB59282		
**Species**	*Stratiomys singularior*		
**BioSample**	SAMEA11025046		
**NCBI taxonomy ID**	511597		
Specimen information			
**Technology**	**ToLID**	**BioSample accession**	**Organism part**
**PacBio long read sequencing**	idStrSing1	SAMEA11025303	thorax
**Hi-C sequencing**	idStrSing1	SAMEA11025314	head
**RNA sequencing**	idStrSing1	SAMEA11025303	thorax
Sequencing information			
**Platform**	**Run accession**	**Read count**	**Base count (Gb)**
**Hi-C Illumina NovaSeq 6000**	ERR10818313	9.23e+08	139.44
**PacBio Sequel IIe**	ERR10812856	1.83e+06	21.27
**RNA Illumina NovaSeq 6000**	ERR11641123	8.67e+07	13.09

**Figure 2.**
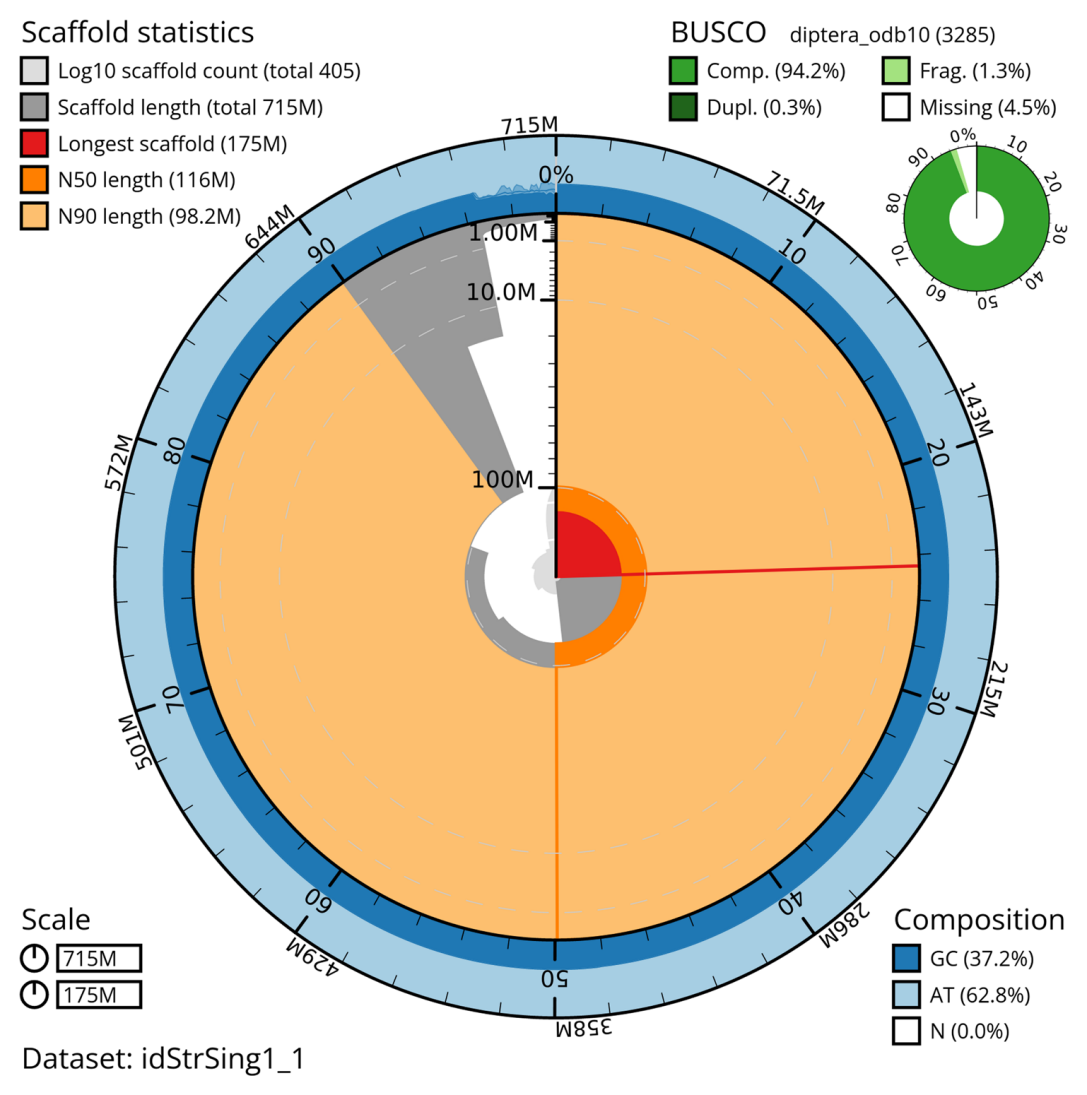
Genome assembly of *Stratiomys singularior*, idStrSing1.1: metrics. The BlobToolKit snail plot shows N50 metrics and BUSCO gene completeness. The main plot is divided into 1,000 size-ordered bins around the circumference with each bin representing 0.1% of the 715,212,284 bp assembly. The distribution of scaffold lengths is shown in dark grey with the plot radius scaled to the longest scaffold present in the assembly (175,337,657 bp, shown in red). Orange and pale-orange arcs show the N50 and N90 scaffold lengths (116,479,916 and 98,237,331 bp), respectively. The pale grey spiral shows the cumulative scaffold count on a log scale with white scale lines showing successive orders of magnitude. The blue and pale-blue area around the outside of the plot shows the distribution of GC, AT and N percentages in the same bins as the inner plot. A summary of complete, fragmented, duplicated and missing BUSCO genes in the diptera_odb10 set is shown in the top right. An interactive version of this figure is available at https://blobtoolkit.genomehubs.org/view/idStrSing1_1/dataset/idStrSing1_1/snail.

**Figure 3.**
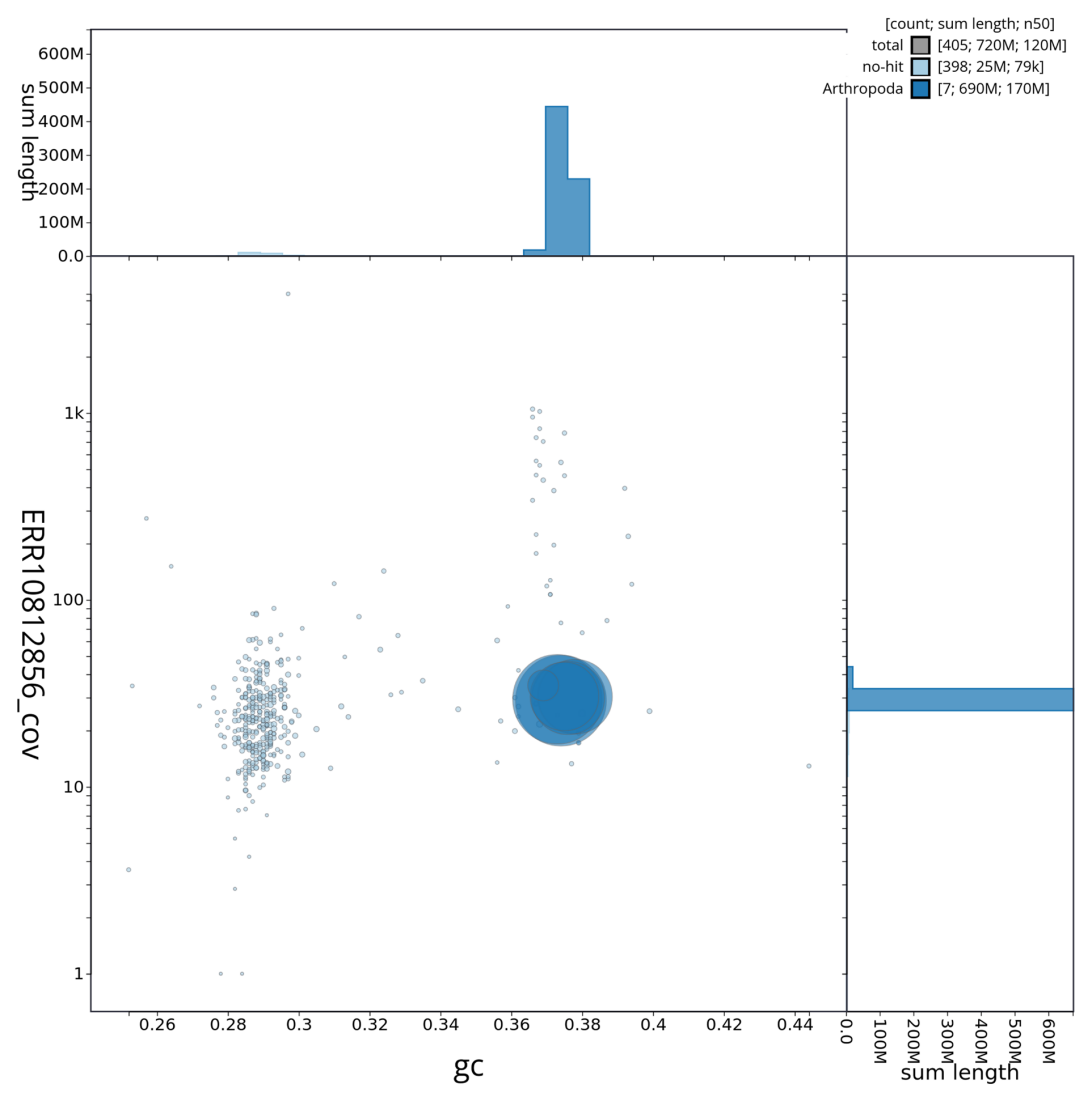
Genome assembly of *Stratiomys singularior*, idStrSing1.1: BlobToolKit GC-coverage plot showing sequence coverage (vertical axis) and GC content (horizontal axis). The circles represent scaffolds, with the size proportional to scaffold length and the colour representing phylum membership. The histograms along the axes display the total length of sequences distributed across different levels of coverage and GC content. An interactive version of this figure is available at https://blobtoolkit.genomehubs.org/view/idStrSing1_1/dataset/idStrSing1_1/blob.

**Figure 4.**
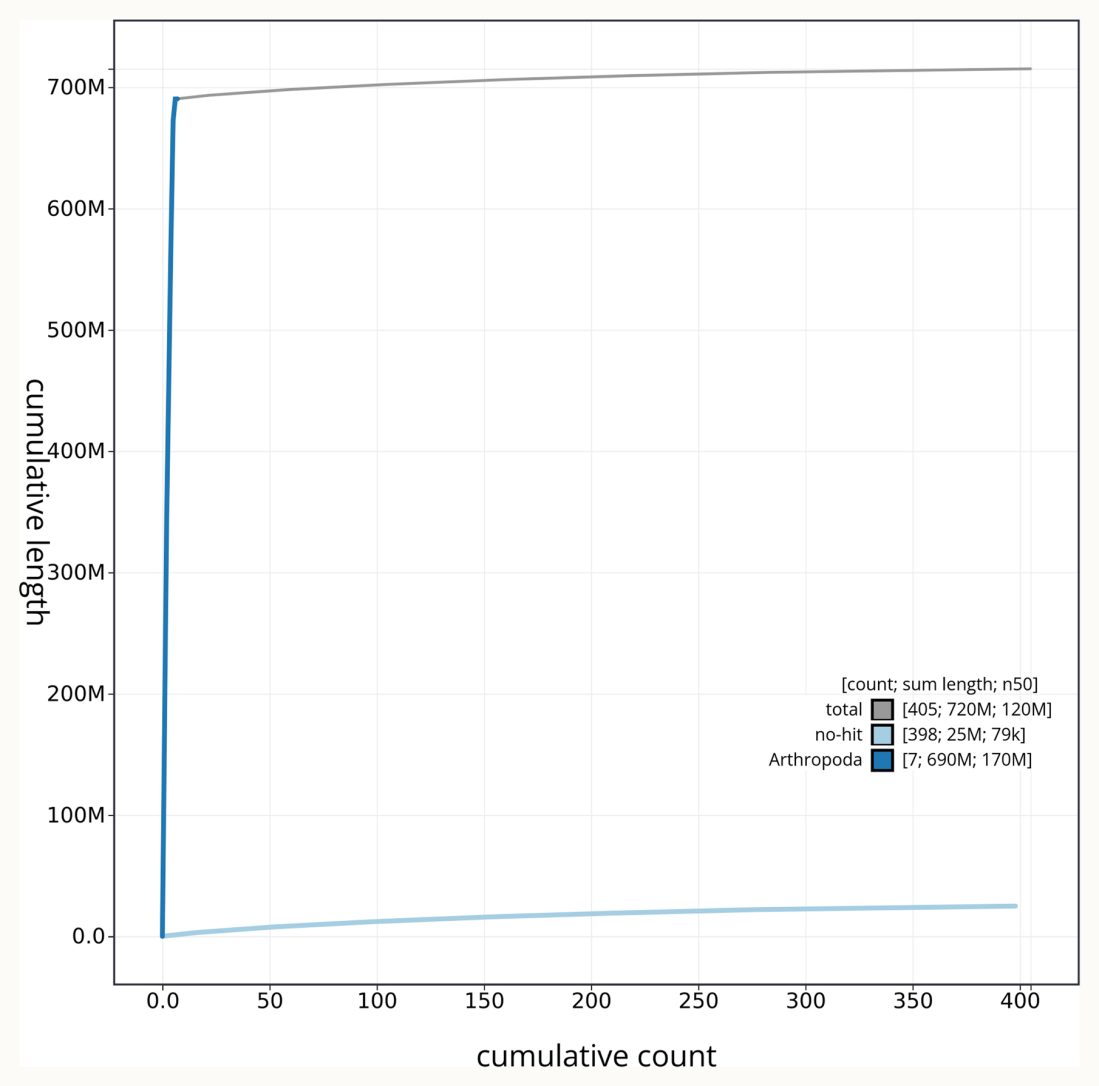
Genome assembly of *Stratiomys singularior* idStrSing1.1: BlobToolKit cumulative sequence plot. The grey line shows cumulative length for all sequences. Coloured lines show cumulative lengths of sequences assigned to each phylum using the buscogenes taxrule. An interactive version of this figure is available at https://blobtoolkit.genomehubs.org/view/idStrSing1_1/dataset/idStrSing1_1/cumulative.

**Figure 5.**
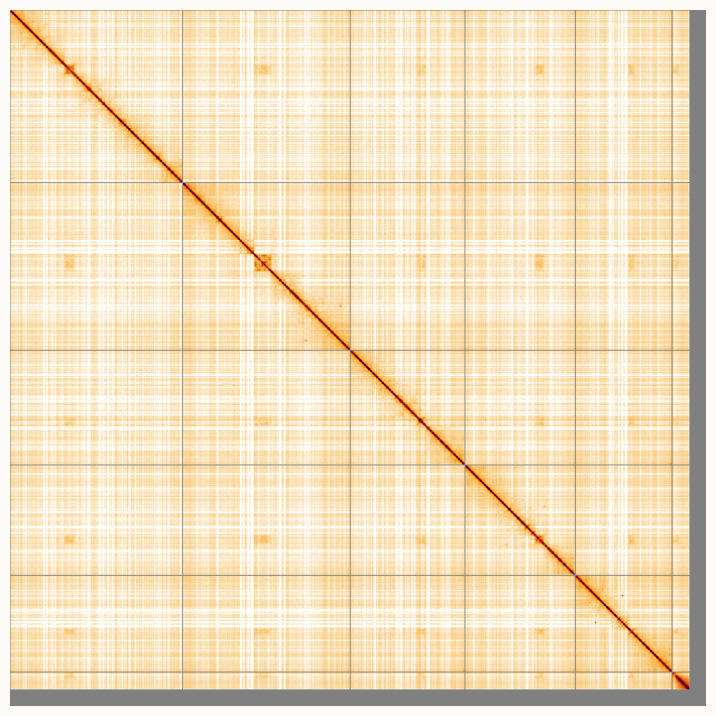
Genome assembly of *Stratiomys singularior*, idStrSing1.1: Hi-C contact map of the idStrSing1.1 assembly, visualised using HiGlass. Chromosomes are shown in order of size from left to right and top to bottom. An interactive version of this figure may be viewed at https://genome-note-higlass.tol.sanger.ac.uk/l/?d=ZTQAaGW9QBGzy01vonmYOQ.

Manual assembly curation corrected 92 missing joins or mis-joins and two haplotypic duplications, reducing the scaffold number by 16.32%, and increasing the scaffold N50 by 3.6%. The final assembly has a total length of 715.20 Mb in 404 sequence scaffolds with a scaffold N50 of 116.5 Mb ( Table 2). The total count of gaps in the scaffolds is 117. The snail plot in Figure 2 provides a summary of the assembly statistics, while the distribution of assembly scaffolds on GC proportion and coverage is shown in Figure 3. The cumulative assembly plot in Figure 4 shows curves for subsets of scaffolds assigned to different phyla. Most (96.56%) of the assembly sequence was assigned to 6 chromosomal-level scaffolds, representing 5 autosomes and the X sex chromosome. Chromosome-scale scaffolds confirmed by the Hi-C data are named in order of size ( Figure 5; Table 3). Chromosome X was assigned based on synteny to *Microchrysa polita* (GCA_949715475.1) ( Crowley *et al.*, 2024). The following regions are of undetermined order and orientation: Chromosome 1 region 55.6–64.8 Mbp, Chromosome 2 region 72.3–89.4 Mbp, Chromosome 3 region 70–74 Mbp, Chromosome 4 region 72.7–81 Mbp and Chromosome 5 region 56.6–59.1 Mbp. While not fully phased, the assembly deposited is of one haplotype. Contigs corresponding to the second haplotype have also been deposited. The mitochondrial genome was also assembled and can be found as a contig within the multifasta file of the genome submission.

**Table 2.** Genome assembly data for *Stratiomys singularior*, idStrSing1.1.

Genome assembly		
Assembly name	idStrSing1.1	
Assembly accession	GCA_954870665.1	
*Accession of alternate haplotype*	*GCA_954871515.1*	
Span (Mb)	715.20	
Number of contigs	522	
Number of scaffolds	404	
Longest scaffold (Mb)	175.71	
Assembly metrics *		*Benchmark*
Contig N50 length (Mb)	36.0	*≥ 1 Mb*
Scaffold N50 length (Mb)	116.5	*= chromosome N50*
Consensus quality (QV)	64.8	*≥ 40*
*k*-mer completeness	100.0%	*≥ 95%*
BUSCO **	C:94.2%[S:93.9%,D:0.3%], F:1.3%,M:4.5%,n:3,285	*S > 90%* *D < 5%*
Percentage of assembly mapped to chromosomes	96.56%	*≥ 90%*
Sex chromosomes	X	*localised homologous pairs*
Organelles	Mitochondrial genome: 15.67 kb	*complete single alleles*
Genome annotation of assembly GCA_954870665.1 at Ensembl		
Number of protein-coding genes	11,614	
Number of non-coding genes	1,360	
Number of gene transcripts	20,327	

* Assembly metric benchmarks are adapted from Rhie *et al.* (2021) and the Earth BioGenome Project Report on Assembly Standards September 2024. ** BUSCO scores based on the diptera_odb10 BUSCO set using version 5.3.2. C = complete [S = single copy, D = duplicated], F = fragmented, M = missing, n = number of orthologues in comparison. A full set of BUSCO scores is available at https://blobtoolkit.genomehubs.org/view/idStrSing1_1/dataset/idStrSing1_1/busco.

**Table 3.** Chromosomal pseudomolecules in the genome assembly of *Stratiomys singularior*, idStrSing1.

INSDC accession	Name	Length (Mb)	GC%
OX940877.1	1	175.34	37.5
OX940878.1	2	170.07	37.5
OX940879.1	3	116.48	38.0
OX940880.1	4	112.19	37.5
OX940881.1	5	98.24	37.5
OX940882.1	X	17.94	37.0
OX940883.1	MT	0.02	30.0

The estimated Quality Value (QV) of the final assembly is 64.8 with *k*-mer completeness of 100.0%, and the assembly has a BUSCO v5.3.2 completeness of 94.2% (single = 93.9%, duplicated = 0.3%), using the diptera_odb10 reference set ( *n* = 3,285).

Metadata for specimens, BOLD barcode results, spectra estimates, sequencing runs, contaminants and pre-curation assembly statistics are given at https://links.tol.sanger.ac.uk/species/511597.

## Genome annotation report

The *Stratiomys singularior* genome assembly (GCA_954870665.1) was annotated at the European Bioinformatics Institute (EBI) on Ensembl Rapid Release. The resulting annotation includes 20,327 transcribed mRNAs from 11,614 protein-coding and 1,360 non-coding genes ( Table 2; https://rapid.ensembl.org/Stratiomys_singularior_GCA_954870665.1/Info/Index). The average transcript length is 18,290.49. There are 1.57 coding transcripts per gene and 5.75 exons per transcript.

## Methods

### Sample acquisition and DNA barcoding

An adult female *Stratiomys singularior* (specimen ID NHMUK014036827, ToLID idStrSing1) was collected from Cothill Fen National Nature Reserve, England, UK (latitude 51.69, longitude –1.33) on 2021-06-19 using an aerial net. The specimen was collected by Duncan Sivell and Olga Sivell (Natural History Museum) and identified by Ryan Mitchell (independent researcher).

The initial identification was verified by an additional DNA barcoding process according to the framework developed by Twyford *et al*. (2024). A small sample was dissected from the specimens and stored in ethanol, while the remaining parts of the specimen were shipped on dry ice to the Wellcome Sanger Institute (WSI). The tissue was lysed, the COI marker region was amplified by PCR, and amplicons were sequenced and compared to the BOLD database, confirming the species identification ( Crowley *et al.*, 2023). Following whole genome sequence generation, the relevant DNA barcode region is also used alongside the initial barcoding data for sample tracking at the WSI ( Twyford *et al.*, 2024). The standard operating procedures for Darwin Tree of Life barcoding have been deposited on protocols.io ( Beasley *et al.*, 2023).

### Nucleic acid extraction

The workflow for high molecular weight (HMW) DNA extraction at the Wellcome Sanger Institute (WSI) Tree of Life Core Laboratory includes a sequence of core procedures: sample preparation and homogenisation, DNA extraction, fragmentation and purification. Detailed protocols are available on protocols.io ( Denton *et al.*, 2023). The idStrSing1 sample was weighed and dissected on dry ice ( Jay *et al.*, 2023) and thorax tissue was cryogenically disrupted using the Covaris cryoPREP ^®^ Automated Dry Pulverizer ( Narváez-Gómez *et al.*, 2023). HMW DNA was extracted using the Automated MagAttract v1 protocol ( Sheerin *et al.*, 2023). DNA was sheared into an average fragment size of 12–20 kb in a Megaruptor 3 system ( Todorovic *et al.*, 2023). Sheared DNA was purified by solid-phase reversible immobilisation, using AMPure PB beads to eliminate shorter fragments and concentrate the DNA ( Strickland *et al.*, 2023). The concentration of the sheared and purified DNA was assessed using a Nanodrop spectrophotometer and Qubit Fluorometer using the Qubit dsDNA High Sensitivity Assay kit. Fragment size distribution was evaluated by running the sample on the FemtoPulse system.

RNA was extracted from thorax tissue of idStrSing1 in the Tree of Life Laboratory at the WSI using the RNA Extraction: Automated MagMax™ *mir*Vana protocol ( do Amaral *et al.*, 2023). The RNA concentration was assessed using a Nanodrop spectrophotometer and a Qubit Fluorometer using the Qubit RNA Broad-Range Assay kit. Analysis of the integrity of the RNA was done using the Agilent RNA 6000 Pico Kit and Eukaryotic Total RNA assay.

### Sequencing

Pacific Biosciences HiFi circular consensus DNA sequencing libraries were constructed according to the manufacturers’ instructions. Poly(A) RNA-Seq libraries were constructed using the NEB Ultra II RNA Library Prep kit. DNA and RNA sequencing was performed by the Scientific Operations core at the WSI on Pacific Biosciences Sequel IIe (HiFi) and Illumina NovaSeq 6000 (RNA-Seq) instruments. Hi-C data were also generated from head tissue of idStrSing1 using the Arima-HiC v2 kit. The Hi-C sequencing was performed using paired-end sequencing with a read length of 150 bp on the Illumina NovaSeq 6000 instrument.

### Genome assembly, curation and evaluation

***Assembly***

The HiFi reads were first assembled using Hifiasm ( Cheng *et al.*, 2021) with the --primary option. Haplotypic duplications were identified and removed using purge_dups ( Guan *et al.*, 2020). The Hi-C reads were mapped to the primary contigs using bwa-mem2 ( Vasimuddin *et al.*, 2019). The contigs were further scaffolded using the provided Hi-C data ( Rao *et al.*, 2014) in YaHS ( Zhou *et al.*, 2023) using the --break option. The scaffolded assemblies were evaluated using Gfastats ( Formenti *et al.*, 2022), BUSCO ( Manni *et al.*, 2021) and MERQURY.FK ( Rhie *et al.*, 2020).

The mitochondrial genome was assembled using MitoHiFi ( Uliano-Silva *et al.*, 2023), which runs MitoFinder ( Allio *et al.*, 2020) and uses these annotations to select the final mitochondrial contig and to ensure the general quality of the sequence.

***Assembly curation***

The assembly was decontaminated using the Assembly Screen for Cobionts and Contaminants (ASCC) pipeline (article in preparation). Flat files and maps used in curation were generated in TreeVal ( Pointon *et al.*, 2023). Manual curation was primarily conducted using PretextView ( Harry, 2022), with additional insights provided by JBrowse2 ( Diesh *et al.*, 2023) and HiGlass ( Kerpedjiev *et al.*, 2018). Scaffolds were visually inspected and corrected as described by Howe *et al*. (2021). Any identified contamination, missed joins, and mis-joins were corrected, and duplicate sequences were tagged and removed. The sex chromosome was identified by synteny analysis. The process is documented at https://gitlab.com/wtsi-grit/rapid-curation (article in preparation).

***Evaluation of the final assembly***

A Hi-C map for the final assembly was produced using bwa-mem2 ( Vasimuddin *et al.*, 2019) in the Cooler file format ( Abdennur & Mirny, 2020). To assess the assembly metrics, the *k*-mer completeness and QV consensus quality values were calculated in Merqury ( Rhie *et al.*, 2020). This work was done using Nextflow ( Di Tommaso *et al.*, 2017) DSL2 pipelines “sanger-tol/readmapping” ( Surana *et al.*, 2023a) and “sanger-tol/genomenote” ( Surana *et al.*, 2023b). The genome was analysed within the BlobToolKit environment ( Challis *et al.*, 2020) and BUSCO scores ( Manni *et al.*, 2021; Simão *et al.*, 2015) were calculated. The genome evaluation pipelines were developed using nf-core tooling ( Ewels *et al.*, 2020) and MultiQC ( Ewels *et al.*, 2016), relying on the Conda package manager, the Bioconda initiative ( Grüning *et al.*, 2018), the Biocontainers infrastructure ( da Veiga Leprevost *et al.*, 2017), as well as the Docker ( Merkel, 2014) and Singularity ( Kurtzer *et al.*, 2017) containerisation solutions.

Table 4 contains a list of relevant software tool versions and sources.

**Table 4.** Software tools: versions and sources.

Software tool	Version	Source
BlobToolKit	4.2.1	https://github.com/blobtoolkit/blobtoolkit
BUSCO	5.3.2	https://gitlab.com/ezlab/busco
bwa-mem2	2.2.1	https://github.com/bwa-mem2/bwa-mem2
Cooler	0.8.11	https://github.com/open2c/cooler
Hifiasm	0.16.1-r375	https://github.com/chhylp123/hifiasm
HiGlass	1.11.6	https://github.com/higlass/higlass
Merqury	MerquryFK	https://github.com/thegenemyers/MERQURY.FK
MitoHiFi	2	https://github.com/marcelauliano/MitoHiFi
Nextflow	23.04.0-5857	https://github.com/nextflow-io/nextflow
PretextView	0.2	https://github.com/wtsi-hpag/PretextView
purge_dups	1.2.3	https://github.com/dfguan/purge_dups
sanger-tol/ascc	-	https://github.com/sanger-tol/ascc
sanger-tol/genomenote	v1.0	https://github.com/sanger-tol/genomenote
sanger-tol/readmapping	1.1.0	https://github.com/sanger-tol/readmapping/tree/1.1.0
YaHS	1.2a	https://github.com/c-zhou/yahs

### Genome annotation

The Ensembl Genebuild annotation system ( Aken *et al.*, 2016) was used to generate annotation for the *Stratiomys singularior* assembly (GCA_954870665.1) in Ensembl Rapid Release at the EBI. Annotation was created primarily through alignment of transcriptomic data to the genome, with gap filling via protein-to-genome alignments of a select set of proteins from UniProt ( UniProt Consortium, 2019).

### Wellcome Sanger Institute – Legal and Governance

The materials that have contributed to this genome note have been supplied by a Darwin Tree of Life Partner. The submission of materials by a Darwin Tree of Life Partner is subject to the **‘Darwin Tree of Life Project Sampling Code of Practice’**, which can be found in full on the Darwin Tree of Life website here. By agreeing with and signing up to the Sampling Code of Practice, the Darwin Tree of Life Partner agrees they will meet the legal and ethical requirements and standards set out within this document in respect of all samples acquired for, and supplied to, the Darwin Tree of Life Project.

Further, the Wellcome Sanger Institute employs a process whereby due diligence is carried out proportionate to the nature of the materials themselves, and the circumstances under which they have been/are to be collected and provided for use. The purpose of this is to address and mitigate any potential legal and/or ethical implications of receipt and use of the materials as part of the research project, and to ensure that in doing so we align with best practice wherever possible. The overarching areas of consideration are:

•   Ethical review of provenance and sourcing of the material

•   Legality of collection, transfer and use (national and international)

Each transfer of samples is further undertaken according to a Research Collaboration Agreement or Material Transfer Agreement entered into by the Darwin Tree of Life Partner, Genome Research Limited (operating as the Wellcome Sanger Institute), and in some circumstances other Darwin Tree of Life collaborators.

## Data Availability

European Nucleotide Archive: *Stratiomys singularior*. Accession number PRJEB59282; https://identifiers.org/ena.embl/PRJEB59282 ( Wellcome Sanger Institute, 2023). The genome sequence is released openly for reuse. The *Stratiomys singularior* genome sequencing initiative is part of the Darwin Tree of Life (DToL) project. All raw sequence data and the assembly have been deposited in INSDC databases. Raw data and assembly accession identifiers are reported in Table 1 and Table 2.

## Author information

Members of the Natural History Museum Genome Acquisition Lab are listed here: https://doi.org/10.5281/zenodo.12159242.

Members of the Darwin Tree of Life Barcoding collective are listed here: https://doi.org/10.5281/zenodo.12158331

Members of the Wellcome Sanger Institute Tree of Life Management, Samples and Laboratory team are listed here: https://doi.org/10.5281/zenodo.12162482.

Members of Wellcome Sanger Institute Scientific Operations: Sequencing Operations are listed here: https://doi.org/10.5281/zenodo.12165051.

Members of the Wellcome Sanger Institute Tree of Life Core Informatics team are listed here: https://doi.org/10.5281/zenodo.12160324.

Members of the Tree of Life Core Informatics collective are listed here: https://doi.org/10.5281/zenodo.5013541.

Members of the Darwin Tree of Life Consortium are listed here: https://doi.org/10.5281/zenodo.4783558.

## References

[ref-1] Abdennur N Mirny LA : Cooler: scalable storage for Hi-C data and other genomically labeled arrays. *Bioinformatics.* 2020;36(1):311–316. 10.1093/bioinformatics/btz540 31290943 PMC8205516

[ref-2] Aken BL Ayling S Barrell D : The ensembl gene annotation system. *Database (Oxford).* 2016;2016: baw093. 10.1093/database/baw093 27337980 PMC4919035

[ref-3] Allio R Schomaker‐Bastos A Romiguier J : MitoFinder: efficient automated large‐scale extraction of mitogenomic data in target enrichment phylogenomics. *Mol Ecol Resour.* 2020;20(4):892–905. 10.1111/1755-0998.13160 32243090 PMC7497042

[ref-4] Beasley J Uhl R Forrest LL : DNA barcoding SOPs for the Darwin Tree of Life project. *protocols.io.* 2023; [Accessed 25 June 2024]. 10.17504/protocols.io.261ged91jv47/v1

[ref-5] Challis R Richards E Rajan J : BlobToolKit – interactive quality assessment of genome assemblies. *G3 (Bethesda).* 2020;10(4):1361–1374. 10.1534/g3.119.400908 32071071 PMC7144090

[ref-6] Cheng H Concepcion GT Feng X : Haplotype-resolved *de novo* assembly using phased assembly graphs with hifiasm. *Nat Methods.* 2021;18(2):170–175. 10.1038/s41592-020-01056-5 33526886 PMC7961889

[ref-7] Crowley L Allen H Barnes I : A sampling strategy for genome sequencing the British terrestrial arthropod fauna [version 1; peer review: 2 approved]. *Wellcome Open Res.* 2023;8:123. 10.12688/wellcomeopenres.18925.1 37408610 PMC10318377

[ref-8] Crowley LM, University of Oxford and Wytham Woods Genome Acquisition Lab, Darwin Tree of Life Barcoding collective : The genome sequence of the black-horned Gem soldier fly *Microchrysa polita.* (Linnaeus, 1758) [version 1; peer review: 1 approved]. *Wellcome Open Res.* 2024;9:569. 10.12688/wellcomeopenres.23075.1

[ref-9] da Veiga Leprevost F Grüning BA Alves Aflitos S : BioContainers: an open-source and community-driven framework for software standardization. *Bioinformatics.* 2017;33(16):2580–2582. 10.1093/bioinformatics/btx192 28379341 PMC5870671

[ref-10] Denton A Yatsenko H Jay J : Sanger Tree of Life wet laboratory protocol collection V.1. *protocols.io.* 2023. 10.17504/protocols.io.8epv5xxy6g1b/v1

[ref-11] Di Tommaso P Chatzou M Floden EW : Nextflow enables reproducible computational workflows. *Nat Biotechnol.* 2017;35(4):316–319. 10.1038/nbt.3820 28398311

[ref-12] Diesh C Stevens GJ Xie P : JBrowse 2: a modular genome browser with views of synteny and structural variation. *Genome Biol.* 2023;24(1): 74. 10.1186/s13059-023-02914-z 37069644 PMC10108523

[ref-13] do Amaral RJV Bates A Denton A : Sanger Tree of Life RNA extraction: automated MagMax ^™^ mirVana. *protocols.io.* 2023. 10.17504/protocols.io.6qpvr36n3vmk/v1

[ref-14] Drake M : Aquatic Stratiomyidae (Diptera) on grazing marshes. *Dipterists Digest.* 2005;12:87–90. Reference Source

[ref-15] Ewels P Magnusson M Lundin S : MultiQC: summarize analysis results for multiple tools and samples in a single report. *Bioinformatics.* 2016;32(19):3047–3048. 10.1093/bioinformatics/btw354 27312411 PMC5039924

[ref-16] Ewels PA Peltzer A Fillinger S : The nf-core framework for community-curated bioinformatics pipelines. *Nat Biotechnol.* 2020;38(3):276–278. 10.1038/s41587-020-0439-x 32055031

[ref-17] Formenti G Abueg L Brajuka A : Gfastats: conversion, evaluation and manipulation of genome sequences using assembly graphs. *Bioinformatics.* 2022;38(17):4214–4216. 10.1093/bioinformatics/btac460 35799367 PMC9438950

[ref-18] Grüning B Dale R Sjödin A : Bioconda: sustainable and comprehensive software distribution for the life sciences. *Nat Methods.* 2018;15(7):475–476. 10.1038/s41592-018-0046-7 29967506 PMC11070151

[ref-19] Guan D McCarthy SA Wood J : Identifying and removing haplotypic duplication in primary genome assemblies. *Bioinformatics.* 2020;36(9):2896–2898. 10.1093/bioinformatics/btaa025 31971576 PMC7203741

[ref-20] Harry E : PretextView (Paired REad TEXTure Viewer): a desktop application for viewing pretext contact maps.2022. Reference Source

[ref-21] Howe K Chow W Collins J : Significantly improving the quality of genome assemblies through curation. *GigaScience.* 2021;10(1): giaa153. 10.1093/gigascience/giaa153 33420778 PMC7794651

[ref-22] Jay J Yatsenko H Narváez-Gómez JP : Sanger Tree of Life sample preparation: triage and dissection. *protocols.io.* 2023. 10.17504/protocols.io.x54v9prmqg3e/v1

[ref-23] Kerpedjiev P Abdennur N Lekschas F : HiGlass: web-based visual exploration and analysis of genome interaction maps. *Genome Biol.* 2018;19(1): 125. 10.1186/s13059-018-1486-1 30143029 PMC6109259

[ref-24] Kurtzer GM Sochat V Bauer MW : Singularity: scientific containers for mobility of compute. *PLoS One.* 2017;12(5): e0177459. 10.1371/journal.pone.0177459 28494014 PMC5426675

[ref-25] Manni M Berkeley MR Seppey M : BUSCO update: novel and streamlined workflows along with broader and deeper phylogenetic coverage for scoring of eukaryotic, prokaryotic, and viral genomes. *Mol Biol Evol.* 2021;38(10):4647–4654. 10.1093/molbev/msab199 34320186 PMC8476166

[ref-26] Merkel D : Docker: lightweight Linux containers for consistent development and deployment. *Linux J.* 2014;2014(239):2, [Accessed 2 April 2024]. Reference Source

[ref-27] Narváez-Gómez JP Mbye H Oatley G : Sanger Tree of Life sample homogenisation: Covaris cryoPREP ^®^ automated Dry Pulverizer V.1. *protocols.io.* 2023. 10.17504/protocols.io.eq2lyjp5qlx9/v1

[ref-28] NBN Trust: *Stratiomys singularior* (Harris, 1778) map on the NBN Atlas.2024. Reference Source

[ref-29] Pointon DL Eagles W Sims Y : sanger-tol/treeval v1.0.0 – Ancient Atlantis.2023. 10.5281/zenodo.10047654

[ref-30] Rao SSP Huntley MH Durand NC : A 3D map of the human genome at kilobase resolution reveals principles of chromatin looping. *Cell.* 2014;159(7):1665–1680. 10.1016/j.cell.2014.11.021 25497547 PMC5635824

[ref-31] Rhie A McCarthy SA Fedrigo O : Towards complete and error-free genome assemblies of all vertebrate species. *Nature.* 2021;592(7856):737–746. 10.1038/s41586-021-03451-0 33911273 PMC8081667

[ref-32] Rhie A Walenz BP Koren S : Merqury: reference-free quality, completeness, and phasing assessment for genome assemblies. *Genome Biol.* 2020;21(1): 245. 10.1186/s13059-020-02134-9 32928274 PMC7488777

[ref-33] Rozkošný R : A biosystematic study of the European Stratiomyidae (Diptera). Vol. 1. Introduction, Beridinae, Sarginae & Stratiomyidae. The Hague: W. Junk,1982;21. Reference Source

[ref-34] Sheerin E Sampaio F Oatley G : Sanger Tree of Life HMW DNA extraction: automated plant MagAttract v.1. *protocols.io.* 2023. 10.17504/protocols.io.x54v9p2z1g3e/v1

[ref-35] Simão FA Waterhouse RM Ioannidis P : BUSCO: assessing genome assembly and annotation completeness with single-copy orthologs. *Bioinformatics.* 2015;31(19):3210–3212. 10.1093/bioinformatics/btv351 26059717

[ref-36] Strickland M Cornwell C Howard C : Sanger Tree of Life fragmented DNA clean up: manual SPRI. *protocols.io.* 2023. 10.17504/protocols.io.kxygx3y1dg8j/v1

[ref-37] Stubbs A Drake M : British Soldierflies and their Allies. 2nd Edition. British Entomological and Natural History Society,2014. Reference Source

[ref-38] Surana P Muffato M Qi G : sanger-tol/readmapping: sanger-tol/readmapping v1.1.0 - Hebridean Black (1.1.0). *Zenodo.* 2023a. 10.5281/zenodo.7755669

[ref-39] Surana P Muffato M Sadasivan Baby C : sanger-tol/genomenote (v1.0.dev). *Zenodo.* 2023b. 10.5281/zenodo.6785935

[ref-40] Todorovic M Sampaio F Howard C : Sanger Tree of Life HMW DNA fragmentation: diagenode Megaruptor ^®^3 for PacBio HiFi. *protocols.io.* 2023. 10.17504/protocols.io.8epv5x2zjg1b/v1

[ref-41] Twyford AD Beasley J Barnes I : A DNA barcoding framework for taxonomic verification in the Darwin Tree of Life project [version 1; peer review: 2 approved]. *Wellcome Open Res.* 2024;9:339. 10.12688/wellcomeopenres.21143.1 39386966 PMC11462125

[ref-42] Uliano-Silva M Ferreira JGRN Krasheninnikova K : MitoHiFi: a python pipeline for mitochondrial genome assembly from PacBio high fidelity reads. *BMC Bioinformatics.* 2023;24(1): 288. 10.1186/s12859-023-05385-y 37464285 PMC10354987

[ref-43] UniProt Consortium: UniProt: a worldwide hub of protein knowledge. *Nucleic Acids Res.* 2019;47(D1):D506–D515. 10.1093/nar/gky1049 30395287 PMC6323992

[ref-44] Vasimuddin M Misra S Li H : Efficient architecture-aware acceleration of BWA-MEM for multicore systems. In: *2019 IEEE International Parallel and Distributed Processing Symposium (IPDPS)*. IEEE,2019;314–324. 10.1109/IPDPS.2019.00041

[ref-45] Wellcome Sanger Institute: The genome sequence of a soliderfly, *Stratiomys singularior* (Harris, 1776), European Nucleotide Archive, [dataset], accession number PRJEB59282,2023.

[ref-46] Woodley NE : A world catalog of the Stratiomyidae (Insecta: Diptera). North American Dipterists’ Society,2001;11. Reference Source

[ref-47] Zeegers T Schulten A : Families of flies with three pulvilli: fieldguide to northwest Europe. ‘s Graveland: Stichting Jeugdbondsuitgeverij,2022. Reference Source

[ref-48] Zhou C McCarthy SA Durbin R : YaHS: yet another Hi-C scaffolding tool. *Bioinformatics.* 2023;39(1): btac808. 10.1093/bioinformatics/btac808 36525368 PMC9848053

