# Supplementary material for: The genome sequence of a soliderfly, Stratiomys singularior (Harris, 1776)
Source: Wellcome Open Res. 2024 Nov 5;9:646. [Version 1] doi: 10.12688/wellcomeopenres.23294.1 (PMC11599808; doi:10.12688/wellcomeopenres.23294.1)
Supplement: Peer review history

# Reviewer report: Reviewer response for version 1

**DOI:** 10.21956/wellcomeopenres.25676.r111608

**Published:** 2024-11-27

**Authors:** Arun Arumugaperumal

**Affiliations:** 1 Department of Biotechnology, Rajalakshmi Engineering College, Thandalam 602105, Chennai, Tamil Nadu, India

This is the first report of the genome sequence of *Stratiomys singularior*. The assembly reported here is of size 715.2 Mb. The authors have assembled the mitochondrial genome and used Ensembl to identify 11,614 protein coding genes in this assembly.

 The authors have used long read sequencing technique to arrive at a high  quality genome assembly. BUSCO completeness of 94 % is a good indication of it. 

 The data article can be accepted for indexing.

Are sufficient details of methods and materials provided to allow replication by others?

Yes

Is the rationale for creating the dataset(s) clearly described?

Yes

Are the datasets clearly presented in a useable and accessible format?

Yes

Are the protocols appropriate and is the work technically sound?

Yes

Reviewer Expertise:

Bioinformatics; Genomics

I confirm that I have read this submission and believe that I have an appropriate level of expertise to confirm that it is of an acceptable scientific standard.

# Reviewer report: Reviewer response for version 1

**DOI:** 10.21956/wellcomeopenres.25676.r111614

**Published:** 2024-11-26

**Authors:** Zexi Cai

**Affiliations:** 1 Aarhus University, Aarhus, Denmark

The authors has presented a high quality assembly of *Stratiomys singularior*. The charestics of the species with the assembled and annotated genome was well described. The method was also well described. The figures in the manuscript can show the audien the quality of the assembly. The part of the manul curation of the assembly is very valuable.

Are sufficient details of methods and materials provided to allow replication by others?

Yes

Is the rationale for creating the dataset(s) clearly described?

Yes

Are the datasets clearly presented in a useable and accessible format?

Yes

Are the protocols appropriate and is the work technically sound?

Yes

Reviewer Expertise:

Animal genetics and genomics.

I confirm that I have read this submission and believe that I have an appropriate level of expertise to confirm that it is of an acceptable scientific standard.

# Reviewer report: Reviewer response for version 1

**DOI:** 10.21956/wellcomeopenres.25676.r111613

**Published:** 2024-11-19

**Authors:** Anthony Bayega

**Affiliations:** 1 McGill University, Montreal, Quebec, Canada

Sivell et al. provide an overview description of the genome of *Stratiomys singularior *which they sequenced and assembled. I commend them for their efforts. My comments follow below:

 1. In Figure 3, the label on the Y-axis which reads “ERR1…” could be changed to something more informative to help the reader make sense of this figure.

 2. Line 7 of the background should end with 'using a'. The 'a' is missing.

 3. The phrase "S. singularior is on the wing from late May to early September, peaking in July" did not sound clear to me.

 Overall, the authors provide a high-quality genome and also assign much of it to chromosomes. Although much work remains to order the scaffolds and complete the gaps and also structurally and functionally annotate the genome, the current work will indeed be valuable to the whole community.

 I therefore recommend the indexing of this genome so that this resource becomes widely accessible to the scientific community.

Are sufficient details of methods and materials provided to allow replication by others?

Yes

Is the rationale for creating the dataset(s) clearly described?

Yes

Are the datasets clearly presented in a useable and accessible format?

Yes

Are the protocols appropriate and is the work technically sound?

Yes

Reviewer Expertise:

Genomics

I confirm that I have read this submission and believe that I have an appropriate level of expertise to confirm that it is of an acceptable scientific standard.
